# Optimized protocol for the generation of an orthotopic colon cancer mouse model and metastasis

**DOI:** 10.1016/j.xpro.2022.102022

**Published:** 2023-01-12

**Authors:** Sophie Richon, Olivier Zajac, Carlos Perez Gonzalez, Danijela Matic Vignjevic

**Affiliations:** 1 Institut Curie, PSL Research University, CNRS UMR 144, 75005 Paris, France

**Keywords:** Cell Biology, Cancer, Health Sciences, Microscopy, Model Organisms, Organoids

## Abstract

The microenvironment plays an essential role in tumor development and metastatic progression. Here, we describe a simple and rapid protocol to generate tumors in mice using colon cancer cell lines or tumoroids in the correct microenvironment, colonic mucosa. We also detail steps for monitoring the growth of the primary tumor in real time using colonoscopy or *in vivo* imaging system, as well as monitoring metastasis development. Finally, we describe tissue collection and sample preparation for subsequent immunohistochemistry analysis.

## Highlights

• Mechanical injury of colonic mucosa to facilitate cell engraftment
• Injection of colon cancer cells or tumoroids in the colon lumen of mice
• Growth of the orthotopic primary tumor followed by endoscopy or intravital imaging
• Analysis of primary colon tumor and metastasis

## Abstract (editor highlights)

Publisher’s note: Undertaking any experimental protocol requires adherence to local institutional guidelines for laboratory safety and ethics.

## Graphical abstract

**Figure:**
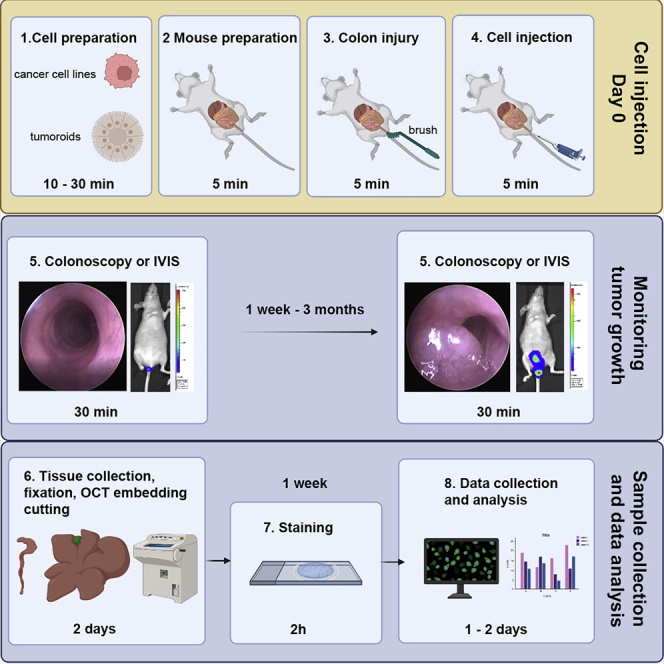

## Before you begin

The tumor microenvironment plays critical roles during tumor development, cell dissemination and metastasis formation. The development of colon cancer in the correct microenvironment could be studied using transgenic animals or carcinogen/inflammation-induced tumor mouse models. However, these models are slow and rarely recapitulate tumor progression until metastasis. Alternative models include injecting colon cancer cells subcutaneously or in the cecum; however, that does not recapitulate the natural microenvironment for colon cancer cells. While several publications have recently described the transplantation of normal organoids into injured colonic mucosa,^1^^,^^2^ here we describe a protocol that enables the generation of primary colonic tumors that can metastasize into the liver. This rapid and reproducible protocol also significantly reduces mouse mortality due to the procedure.

### Institutional permissions

This protocol requires the use of mice. All mouse experiments should be performed following relevant governmental and institutional guidelines. Animals used in this study were maintained in the SPF animal facility of Institut Curie (Paris, France) before use. All experiments described in this study were performed in accordance with the European and French National Regulation for the Protection of Vertebrate Animals used for Experimental and other Scientific Purposes (2010/63/UE) for the care and use of laboratory animals. All experimental procedures were approved by the ethics committee of the Institut Curie CEEA-IC #118 amendment to the projects 2020-002 (Authorization: #25603-2020053122444776-v2) and 2020-010 (Authorization: #27460-2020100614277480-v1) given by the National Authority in compliance with the international guidelines.

### Expansion of cell lines and organoids

**Timing: 2–7 days (for step 1)**

**Timing: 3–7 days (for step 2)**

The protocol described in this paper is based on commercial human colon cancer cell line SW480 and “in-house” mouse tumor organoids (tumoroids). We have also used this protocol successfully with other colorectal cancer cell lines, such as mouse cell lines CT26 and MC38 and human cell lines HCT116, HT29, and SW837, as well as mouse intestinal organoids from tumor tissue.

1. Preparation of commercial cell lines.
  ***Note:*** We used Dukes' type B colorectal adenocarcinoma SW480 (ATCC CCL-228) cells infected with lentiviruses carrying either plasmid to express GFP (pWXId.xdnaGFP) or luciferase (pLJM1 luciferase).
  a. Culture the cells in DMEM Glutamax medium supplemented with 10% Fetal calf serum in 75 cm^2^ culture flasks until 80% confluence.
  b. Split cells 1:5 twice a week.
    ***Note:*** This is sufficient to inject about 20 mice (0.5 million cells per mouse).

2. Preparation of tumor organoids.
  ***Note:*** We used tumoroids isolated from spontaneously developed intestinal tumors Apc fl/fl+; P53−/−, Kras-G12D/+;Vil1CreERT2;Lgr5DTR/eGFP, R26R-Confetti RFP-CFP.^3^^,^^4^
  ***Note:*** We tried the protocol until 30 passages and did not notice that passage number affects tumor engraftment. The size of tumoroids was between 30–200 μm (see Figure 5A).
  ***Note:*** Before starting the experiment, expand tumoroids to obtain 12 wells of 24-well plate to have 0,5 million cells per mouse.
  a. Detach Matrigel drops by scratching using the tip of a 1 mL pipette.
  b. Aspirate the drop and the medium using a 1 mL pipette.
  c. Transfer to a 15 mL tube and add medium until 10 mL to balance the centrifuge.
  d. Spin down cells for 10 min at 450 g at room temperature.
  e. Aspirate the supernatant.
  f. Add 1 mL of Tryplee Express (room temperature) to the pellet.
  g. Incubate for 3 min at 37°C, 5% CO_2_, until tumoroids dissociation.
  h. Add 5 mL medium with 10% FBS to inactivate Tryplee Express.
  i. Spin down cells for 10 min at 450 g at room temperature.
  j. Aspirate the supernatant.
  k. Resuspend cell pellets in 50:50 Matrigel/tumoroids culture medium mix on ice to prevent premature Matrigel polymerization.
  l. Deposit 50 μL drops at the bottom of the 24-well plate pre-wormed at 37°C to speed up Matrigel polymerization and thus prevent flattening of the drop.
  m. Put the plate upside-down at 37°C, 5% CO_2,_ for 30 min to polymerize Matrigel.
    ***Note:*** The upside-down position prevents the tumoroids from sinking to the bottom of the Matrigel drop and adhering to the plastic.
  n. Add 350 μL of medium to each well.
  o. Put the plate at 37°C, 5% CO_2_. After 3 days, split tumoroids to expand their number.
    ***Note:*** Repeat splitting until the required number of cells per mouse is obtained.

### Mouse housing

**Timing: 7 days**

***Note:*** We graft mouse tumoroids and SW480GFP-LUC cells in Nude NMRI mice. However, for other cell lines, we used mice with different backgrounds to match the background of each cell line. For example, graft MC38 cells in C57BL/6 mice, and CT26 cells in Balb/c mice.

***Note:*** We use six weeks old mice, both male and female. Acclimatize mice in the animal facility for at least one week before injection.

### Preparation of equipment and materials for colon injection

**Timing: 7 days**

***Note:*** The culture media for the tumoroids should be used fresh and thus prepared on the same day when tumoroids are amplified and prepared for injection. The EDTA solution should be placed in a water bath heated to 50°C for 15 min before the injection of the cells.

## Key resources table

**Table:** 

REAGENT or RESOURCE	SOURCE	IDENTIFIER
**Chemicals, peptides, and recombinant proteins**		

DMEM glutamax 4.5 g/l glucose	Gibco	61965.026
Fetal bovine serum (FBS)	Gibco	10270-106
PBS 1× without calcium and magnesium	Pan Biotech	P04-36010P
Trypan blue	Molecular Probes	T10282
DMEM-F12 - Glutamax	Gibco	10565-018
Anti/Anti 100×	Gibo	15240-062
Noggin	Peprotech	250-38
EGF	Peprotech	315-09
mbFGF (mouse recombinant)	Peprotech	450-33
B27 50×	Life Technologies	12587-010
Matrigel	Corning	354234
ISO-VET (isoflurane)	Piramal Critical Care	N/A
Rimadyl 50 mg/mL (carprofen)	Zoetis	N/A
EDTA 0.5 M pH8.0	Invitrogen	15575-020
Gel-Larmes Gel Ophtalmique 10 g	Théa	
Biological glue – Vetbond	Phymep -3M	1469SB
KY Lubricating Jelly Sterile	Reckitt BenckiserHealthcare	N/A
IVISbrite D-Luciferin Potassium Salt Bioluminescent Substrate (XenoLight D-Luciferin, Potassium Salt, 1 g)	PerkinElmer	122 799
KY Lubricating Jelly Sterile	Reckitt Benckiser Healthcare	N/A
Paraformaldehyde 16%	Electron Microscopy Sciences	15710
Sucrose	SIGMA	S0389
OCT	SAKURA	4583
DAPI	Invitrogen	D1306
Aqua-Polymount	Polysciences	18606-20

**Experimental models: Cell lines**		

SW480 GFP-LUC (until passage 30)	Home-made	N/A
SW480 (until passage 30)	ATCC	CCL-228
HT29 (until passage 30)	ATCC	HTB-38
HCT116 (until passage 30)	ATCC	CCL-247
SW837 (until passage 30)	ATCC	CCL-235
MC38 (until passage 30)	O. Lantz, Institut Curie	N/A
CT26 (until passage 30)	ATCC	CRL-2638
Tumoroids (until passage 15)	F. Souvage, Genentech	N/A

**Experimental models: Organisms/strains**		

NMRI mice 5 weeks old female	Janvier Labs Saint Genest – Saint Isle - France	N/A
C57BL/6 mice 5 weeks old female	Charles River Lyon - France	N/A
Balb/c mice 5 weeks old female	Charles River Lyon - France	N/A

**Software and algorithms**		

Fiji	Schindelin et al.^5^	https://imagej.net/software/fiji/downloads
Living Image Software	Caliper Life Sciences	N/A

**Other**		

Disposable base Mold 15∗15∗5 mm	Electron Microscopy Sciences	62352-15
Superfrost Plus Adhesion Microscope Slides	Epredia	J1800AMNZ
Cover Glass 24∗50 mm	VWR	631-0147
Countess Invitrogen	Invitrogen	C10227
Countess cell counting chamber slides Invitrogen	Invitrogen	C10283
Electric toothbrush	Panasonic	EW-DL22
Soft interdental brush (Panasonic EW0945)	Panasonic	EW0945
Water bath	Thermo Scientific	
Endoscope	Storz Mainz	Coloview system
Cryostat	Leica	CM1950
Microscope	Invitrogen	EVOS M5000
Microscope confocal	LSM880 NLO	CLSM
LISPI microscope	NIKON	ECLIPSE Ti2
IVIS	Caliper	Lumina II
Anesteo Tec 7 (anesthesia chamber)	MSS	Tec 7

***Note:*** Soft interdental brush (Panasonic EW0945) **is critical material**.

## Materials and equipment

Recipes are provided to a specified final volume, but investigators may choose to prepare different volumes depending on the number of samples processed.

**Table:** Culture medium for cell lines

Reagent	Final concentration	Stock concentration	Add to 500 mL
DMEM glutamax 4.5 g/l glucose	1×	1×	450 mL
Fetal Bovine Serum (FBS)	10%	100%	50 mL

Store at +4°C for up to 1 month.

**Table:** Culture medium for Tumoroids

Reagent	Final concentration	Stock concentration	Add to 10 mL
DMEM-F12 – Glutamax	1×		9.6 mL
Anti-Anti	1×	100×	100 μL
Noggin	100 ng/mL	0.1 mg/mL	10 μL
EGF	20 ng/mL	0.5 mg/mL	0.4 μL
mbFGF	10 ng/mL	100 μg/mL	1 μL
B27	1×	50×	200 μL

Store at +4°C for 1 week.

**Table:** Solution of Carprofen

Reagent	Final concentration	Stock concentration	Add to 1 mL
Rimadyl	0.5 mg/mL	50 mg/mL	10 μL
PBS	1×	1×	990 μL

Use freshly prepared.

***Note:*** The dose injected per mouse is 5 mg/kg.

**Table:** Luciferin solution

Reagent	Final concentration	Add to 67 mL
Luciferin	15 mg/mL	1 g
PBS	1×	67 mL

Aliquot in 1 mL tube and store at −20°C- protect from light up to 6 months.

**Table:** Paraformaldehyde solution

Reagent	Final concentration	Stock concentration	Add to 40 mL
Paraformaldehyde	4%	16%	10 mL
PBS	1×	1×	30 mL

Store at +4°C one week – Protect from light.

**Table:** 15% Sucrose solution

Reagent	Final concentration	Add to 100 mL
Sucrose	15%	15 g
PBS	1×	100 mL

Store at +4°C up to 1 month.

**Table:** 30% Sucrose solution

Reagent	Final concentration	Add to 100 mL
Sucrose	30%	30 g
PBS	1×	100 mL

Store at +4°C up to 1 month.

## Step-by-step method details

### Preparation of cells for injection (day 0)

**Timing: 10–30 min**

**Timing: 10 min (for step 1)**

**Timing: 30 min (for step 2)**

This section describes the preparation of cells that will be injected into the mouse colon.

***Note:*** We injected 0.5 million cells (SW480 or tumoroids) per mouse.

1. Prepare cells from lines.
  a. Remove cell culture medium by aspiration using a 2 mL pipette.
  b. Wash cells with 5 mL of room temperature PBS.
  c. Detach cells by adding 3 mL of Tryplee Express to a T75 cm^2^ flask.
  d. Incubate cells at 37°C until they detach.
    ***Note:*** It takes approximately 3–5 min, depending on cell type.
  e. Add 15 mL of culture media containing 10% FBS to inactivate Tryplee Express.
  f. Count cells:
    i. Take 10 μL of cell suspension and add 10 μL of trypan blue.
    ii. Deposit this mix on cell counting chamber slides and count cells with cell counter.
    iii. Calculate the volume of cells needed to obtain 0.5 × 10^6^ cells in 100 μL that will be injected in each mouse.
      ***Note:*** Routinely, cell viability is above 95%. If cell viability is less than 80%, do not use these cells.
  g. Spin down cells for 3 min at 231 g.
  h. Discard the supernatant and resuspend cells in 100 μL for each mouse of a solution made of 50:50 culture medium (DMEM without FBS) and Matrigel on ice.
  i. Keep cells on ice until injection into mice.
    ***Note:*** In our experience, 2 h of waiting did not affect tumor engraftment.

2. Prepare cells from tumoroids.
  a. Detach Matrigel drops by scratching using the tip of a 1 mL pipette.
  b. Aspirate the drop and medium using a 1 mL pipette.
  c. Transfer all drops to a 15 mL tube. Then add medium until 10 mL to balance the centrifuge.
  d. Spin down cells for 10 min at 450 g at room temperature.
    ***Note:*** room temperature is in range of 19°C–22°C.
  e. Aspirate the supernatant.
  f. Add 1 mL of room temperature Tryplee Express to the pellet.
  g. Incubate for 3 min at 37°C, 5% CO_2_, then assess under the cell culture microscope that tumoroids are dissociated into single cells or small clusters.
  h. Add 5 mL medium to inactivate Tryplee Express.
  i. Count cells:
    i. Take 10 μL of cell suspension and add 10 μL of trypan blue.
    ii. Deposit this mix on cell counting chamber slides and count cells with cell counter.
    iii. Calculate the volume of cells needed to obtain 0.5 × 10^6^ cells in 100 μL that will be injected in each mouse.
      ***Note:*** If there are small clusters, count the cells in each cluster. If cell viability is less than 80%, do not use these tumoroids.
  j. Spin down cells for 10 min at 450 g at room temperature.
  k. Discard the supernatant and resuspend cells in 100 μL for each mouse of a solution made of 50:50 culture medium (DMEM/F12 without FBS) and Matrigel on ice to prevent premature polymerization of Matrigel.
  l. Keep cells on ice until injection into mice. In our experience, 2 h of waiting did not affect tumor engraftment.

### Preparation of mouse (day 0)

**Timing: 5 min per mouse**

This section describes the preparation of mice before cell injection. The mouse needs to be immobilized and anesthetized. An analgesic is given to mice before the procedure to reduce pain. The colon should be cleaned to remove faces, which is done by gently massaging the mouse’s abdomen.

**Figure 1:**
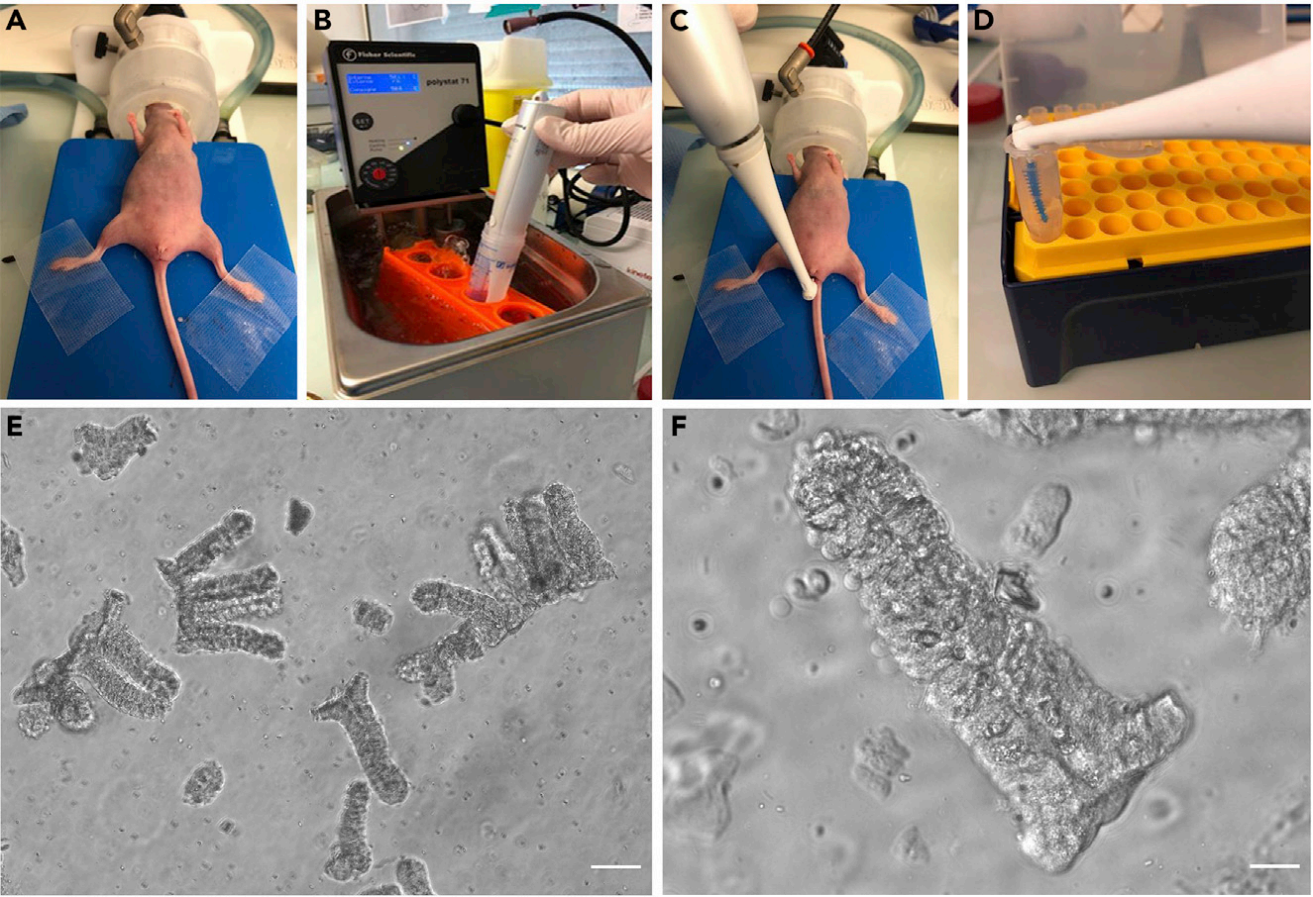
Mouse preparation and crypts removal (A) Anesthetized mouse is placed on its back and kept under isoflurane. The mouse’s legs are immobilized with the tape. (B) The interdental brush is immersed in an EDTA solution heated to 50°C for 30 s. (C) The interdental brush is gently inserted into the mouse’s anus and advanced for 1,5 cm into the colon. (D) The interdental brush is gently removed and immersed in a 1.5 mL Eppendorf tube containing PBS. (E) Removed crypts visualized under EVOS M5000 microscope. Scale bar, 100 μm. (F) High magnification of a crypt isolated from a mouse colon. Scale bar, 25 μm.
8. Remove feces present in the colon by gently massaging the mouse’s abdomen.

***Note:*** You should feel with fingers that the colon is empty.

3. Anesthetize the mouse with 3% isoflurane and 2 L per minute air flow until it falls asleep.
4. Once the mouse is sleeping, reduce isoflurane to 2% with an airflow of 0.5 L per minute.
5. Place tear gel on the eyes of the mice to prevent drying.
6. Inject 5 mg/kg Carprofen (Rimadyl) analgesic (in 100 μL) diluted in PBS subcutaneously.
7. Position the mouse on its back with hind legs apart, immobilized with surgical tape (Figure 1A).

**CRITICAL:** This step is essential because unremoved feces in the distal part of the colon may prevent the cells from attaching to the colonic mucosa. Feces can also open the anus too quickly (which will be obstructed at the end of the experiment using surgical glue) and thus decrease the time of adhesion of cells to the colonic mucosa. Do not wash the colon with PBS or similar solutions, as this will dilute the EDTA and the injected cells.

### Removal of mouse epithelium (day 0)

**Timing: 5 min**

This step injures the colonic mucosa to felicitate the attachment of injected cells. The lesions are made by an electric interdental toothbrush soaked into a hot EDTA solution. The same experimenter can prepare both the mice and EDTA.

9. Pre-heat 0.5 M EDTA, pH8 at 50°C in a water bath 15 min before the start of the experiment.
10. Immerse the interdental brush for 30 s in the pre-heated EDTA solution (Figure 1B).
11. Gently insert the interdental brush into the mouse’s anus and advance it 1.5 cm into the colon (Figure 1C).
12. Turn on the vibration of the interdental brush for 10 s.

**CRITICAL:** This step is crucial because the number of removed crypts must be sufficient but not excessive. The optimal number of detached crypts is between 2,500 and 5,000 crypts. The pressure exerted on the interdental brush should not be too strong to avoid piercing the colonic wall. In case of bleeding, interrupt the abrasions immediately.

13. The interdental brush is gently removed and immersed in an Eppendorf tube containing 1.5 mL of PBS (Figure 1D).
14. Turn on the vibration for 5 s to release the removed crypts (Figures 1E and 1F).
15. Evaluate the efficiency of crypt removal:
  a. Pipette 10 μL of PBS containing crypts on a slide.
  b. Count detached crypts under a cell culture microscope.
  c. Multiply this number by 150 to obtain the total number of detached crypts (that are in 1.5 mL PBS).
16. If there are no or not enough detached crypts, additional abrasions should be performed, assessing the presence of crypts after each abrasion.
17. Eliminate the residual EDTA by massaging the mouse’s abdomen for 2 min.

### Injection of cells in the mouse colon (day 0)

**Timing: 5 min**

At this stage, cells are deposited in the colonic lumen in the area of the lesions made with the interdental brush.

18. Mix the cell solution gently to avoid bubbles.
19. Pipette 100 μL of a solution containing cells and Matrigel using a 200 μL pipette tip.
20. Insert the pipette tip very gently into the colonic lumen parallel to the colon wall and advance as far as possible (about 1.5 cm).
21. Pinch the anus and tip of the pipette with tweezers and slowly pipette out cells into the colonic lumen (Figure 2A).

**Figure 2:**
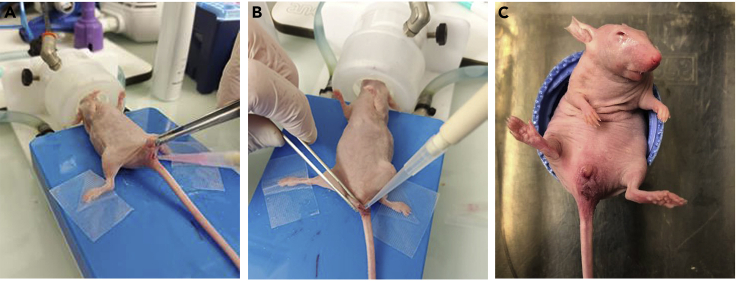
Injection of cells (A) Solution containing cells and Matrigel is inserted in the colonic lumen. (B) A drop of biological glue is placed on the anus, and the edges of the anus are pressed with tweezers to prevent the leakage of the solution containing cells. (C) Mouse is kept with raised pelvis until it wakes up.
22. Gently remove the pipette tip while continue applying pressure to the clamp to keep the anus closed.
23. Carefully remove the cone from the anus while maintaining pressure.
24. Add a drop of surgical glue to close the anus of the mouse (Figure 2B).
25. Hold the mouse in a position with the raised legs and pelvis until it wakes up (Figure 2C).
26. After 4 h, verify that the anus of the mice is opened. If not, remove the remaining glue with a damp tissue.

**CRITICAL:** This step is critical because the cells should not leak from the colonic lumen. The cells should be deposited as far as possible, carefully not to pierce the colon. The volume of deposited glue should be sufficient to hold the anus closed for at least 4 h to allow cell attachment. However, it should not be excessive, ideally self-removed within 4 h.

### Live imaging of tumor growth by endoscopy (day 7 to day 90)

**Timing: 5 min per mouse**

**Figure 3:**
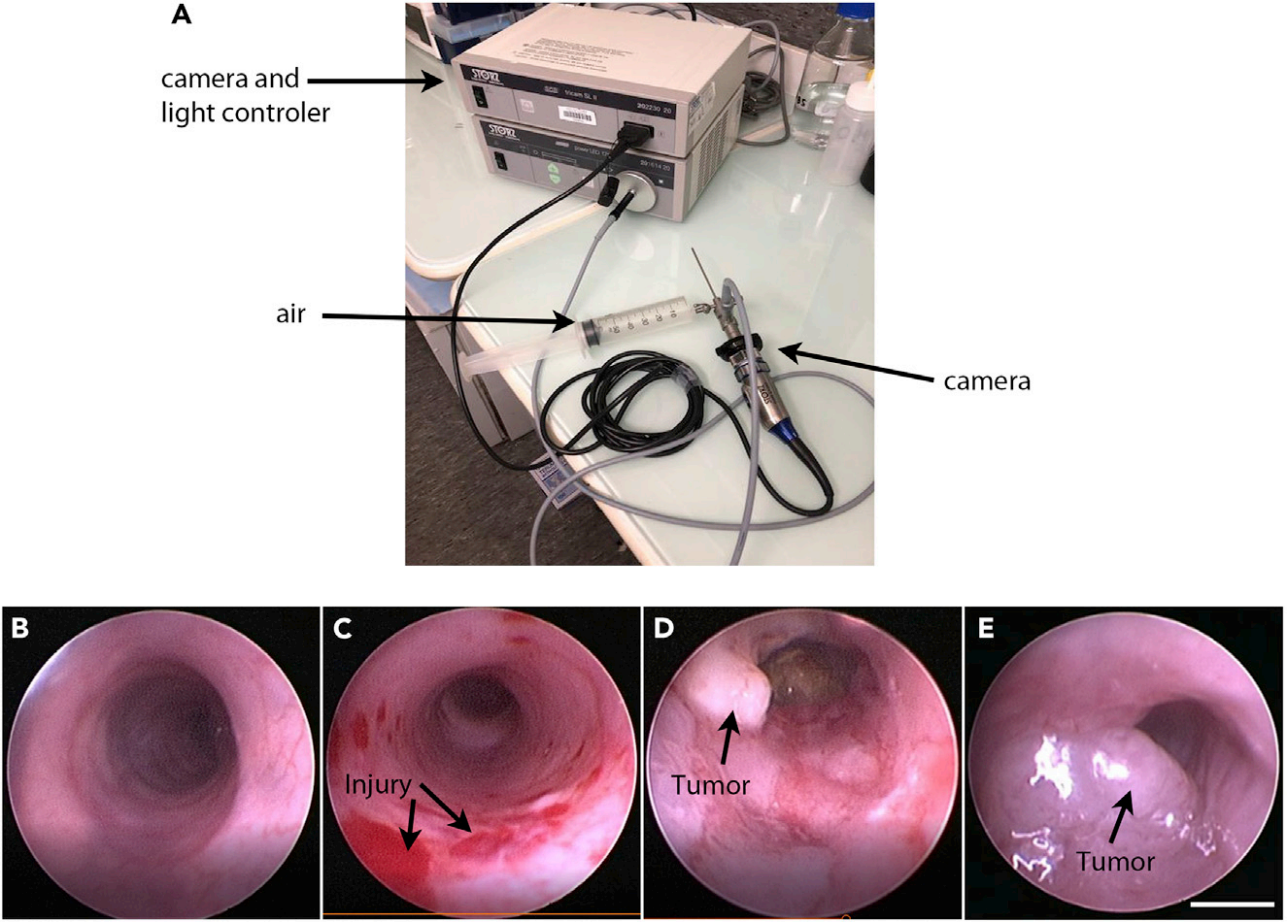
Visualization of tumor growth using endoscopy (A) Animal endoscope composed of a cold light source, a camera control unit, a camera, and a device to inject air to inflate the colon. (B) The colon observed with an endoscope before the procedure. (C) Colon observed after brush injury. (D) Tumor in the colon visualized 15 days after the injury. (E) Tumor in the colon visualized 37 days after the injury. Scale bar, 1 mm.

This step allows visualization of tumor growth by colonoscopy (Figure 3). Colonoscopy could be used to observe a healthy, non-damaged colon (Figure 3B), a colon just after damage with an interdental brush (Figure 3C), or to follow tumor growth (Figures 3D and 3E).

Imaging is done starting one week after the transplantation, once every week and up to 3 months, depending on the tumor growth. Mice could be kept alive until the defined humane endpoint, i.e., 50% obstruction of the colonic lumen or deterioration in the general condition of the animal.

***Note:*** Repeating the imaging several times a week should be avoided to limit repeated anesthesia and limit the risk of bleeding in the animals.

27. Anesthetize mice using 3% isoflurane, 2 L per minute, until it falls asleep.
28. Reduce isoflurane between 2% isoflurane with airflow of 0.5 L per minute during imaging.
29. Immobilize the mouse with tape on the hind legs.
30. Remove feces by abdominal massage as described above.

***Note:*** You should feel with fingers that the colon is empty.

31. Apply vaseline around the anus to facilitate insertion of the endoscope.
32. Insert the endoscope gently into the anus while dilating the colon by blowing air into the port using a 50 mL syringe (Figure 3A). The quantity of air depends on how close the colon is. In general, the injected volume of air is around 5 mL.
33. Move the endoscope throughout the colon gently while recording the movie.

### Live imaging of tumor growth intravital IVIS (day 7 to day 90)

**Timing: 30 min per mouse**

**Figure 4:**
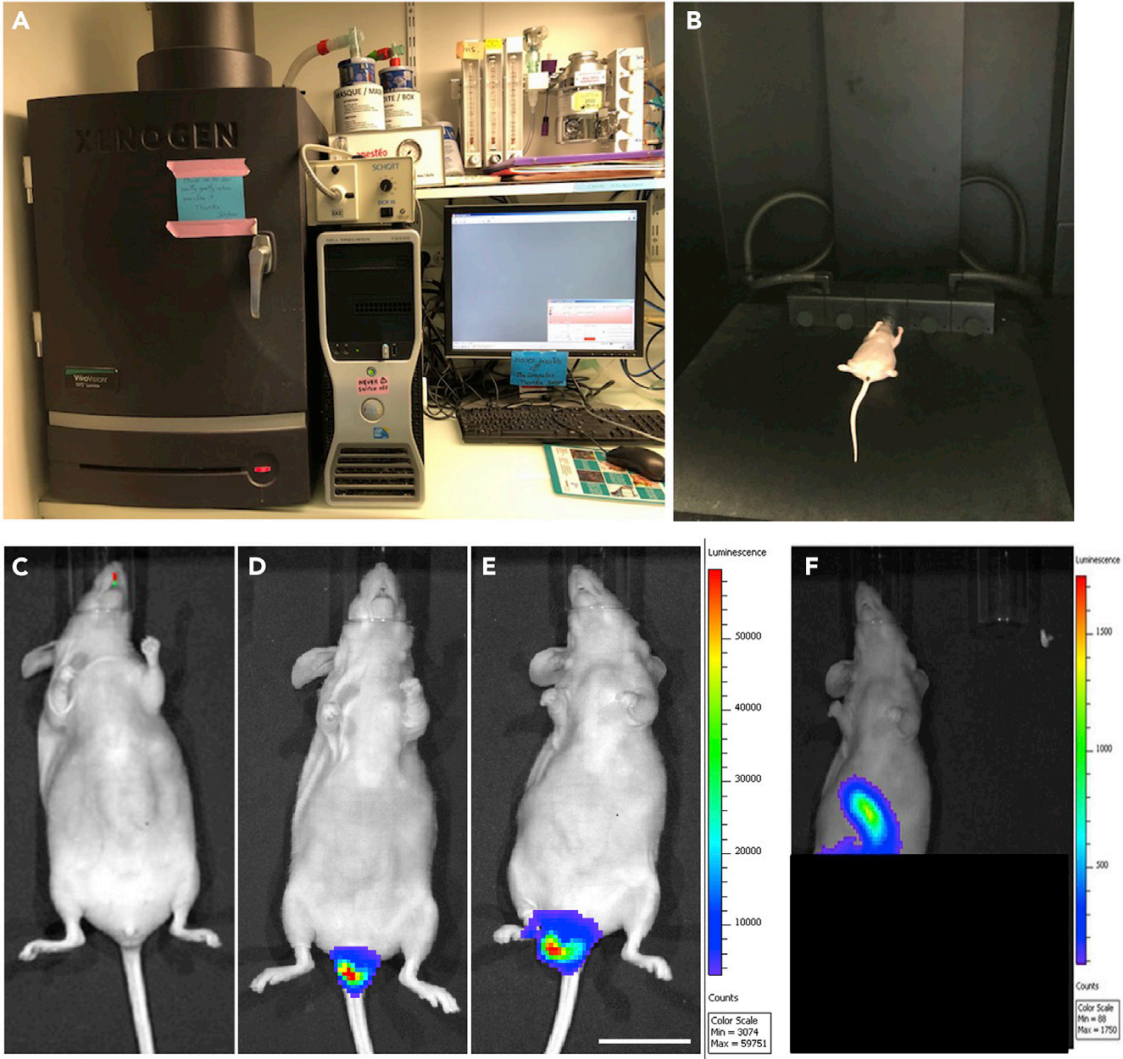
Visualization of tumor growth using IVIS (A) IVIS composed of a CCD camera, an imaging chamber with a heating shelf, and a gas anesthesia manifold. (B) Mouse is placed on its back and kept asleep under isoflurane on the heating shelf after the luciferin injection. Mice are imaged at different time points to follow the tumor growth and emergence of metastases. (C) Mouse before injection of cells. (D) Mouse just after injection of cells. The detected signal is from injected cells. (E) Mouse imaged 37 days after cell injection. The detected signal shows a tumor, Scale bar, 1 cm. (F) Mouse imaged 37 days after cell injection. As the signal from the primary tumor is too strong, obstructing the detection of any potential signal from the metastasis, the mouse’s lower abdomen (colon) is covered with thick paper to block the signal coming from the primary tumor (black square). The detected signal in the upper abdomen represents metastases.

This step allows visualization of tumor growth by IVIS (Figure 4). IVIS could be used to follow the growth of the primary tumor by repeated imaging (Figures 4C–4E) or to visualize metastasis (Figure 4F). Imaging is done starting one week after the transplantation, once every week and up to 3 months, depending on the tumor growth.

***Note:*** Repeating the imaging several times a week should be avoided to limit repeated anesthesia.

34. Inject 200 μL of a 15 mg/mL luciferin solution (150 mg/kg of animal) intraperitoneally.
35. Wait 15 min to ensure consistent photon flux before imaging using IVIS.
36. Anesthetize the mouse with 3% isoflurane and 2 L per minute air flow until it falls asleep.
37. Once the mouse is sleeping, reduce isoflurane to 2% with an airflow of 0.5 L per minute (Figure 4B).
38. Position the mouse on his back in Xenogen Vivovision IVIS lumina II heating chamber (Figures 4A and 4B). Animals are maintained in the instrument using the integral anesthetic manifold. The stage is at a constant temperature of 37°C, which maintains the mouse’s body temperature.
39. Using Living Image software, set up exposure time to 1 min, f-stop to 1 and large pixel binning.

### Collection of samples (day 30 to day 90)

**Timing: 20 min per mouse**

***Note:*** Mice could be sacrificed at a defined time point, but not later than the humane endpoint - when the size of the tumor reaches 50% of the colonic lumen. The colon is systematically removed, while the liver and other organs are removed if macroscopic tumors are detected.

40. Sacrifice mice by cervical dislocation.
41. Open the abdomen and inspect the organs (liver, lungs, peritoneum) for the presence of macroscopic metastases. Macroscopic metastasis could be observed by eyes as white spots (Figure 6B).
42. Cut the urogenital system and pelvis, and then excise the anus and the distal part of the colon (Figure 6A).
43. Inspect colon for presence of tumors.

***Note:*** Tumors could be mistaken by enlarged lymphoid tissues.

44. Take also other organs if they contain macrometastasis.

***Note:*** Metastases will not be formed if primary tumor grow too fast.

### Tissue fixation and embedding (day 30 to day 90)

**Timing: 1 day**

45. Fix organs in 25 mL of 4% paraformaldehyde (PFA) dissolved in PBS using 50 mL tube for 1 h at 4°C with gentle shaking.
46. Decant PFA and wash tissues with PBS for 10 min at 4°C with gentle shaking.
47. Decant PBS and add 15 mL of 15% sucrose in a 50 mL tube at 4°C with gentle shaking. These sucrose steps remove water from the tissues.
48. Incubate tissue for 2 h at 4°C.
49. Decant solution and replace it with 15 mL of 30% sucrose in 50 mL tube.
50. Incubate tissue for 2 h at 4°C.
51. Place tissue in plastic 15 × 15 × 5 mm mold and include in OCT at room temperature. Orient the colon vertically (lumen of the colon facing up) with the anus sitting at the bottom of the mold. For other organs, the orientation of the tissue is not important.
52. Place the mold at −20°C for at least 2 h to let OCT solidify.

### Cutting samples using cryostat (day 30 to day 90)

**Timing: 30 min per sample**

53. Orient the OCT block with the anus/rectum facing the blade.

***Note:*** The OCT block should be trimmed until reaching the tissue.

54. Trim the anus and rectum until reaching the tumor that can be seen macroscopically.
55. Cut colon/tumor into 8 to 10 μm-thick slices.

***Note:*** For other tissues, just trim the OCT and proceed with cutting the tissue into 8 to 10 μm-thick slices.

56. Transfer the tissue to the slide Super frost + by contact.

### Staining samples (day 30 to day 90)

**Timing: 2 h**

57. Add 500 μL of a solution containing 25 μg/mL DAPI in PBS to the sections.
58. Incubate for 30 min at room temperature.
59. Remove the DAPI solution, add 8 drops of Aquapolymount mounting solution on the tissue section and cover with 24 × 50 mm coverslip.
60. Leave to dry for at least 1 h before imaging.

### Imaging samples (day 30 to day 90)

**Timing: 1 h per sample**

**Figure 5:**
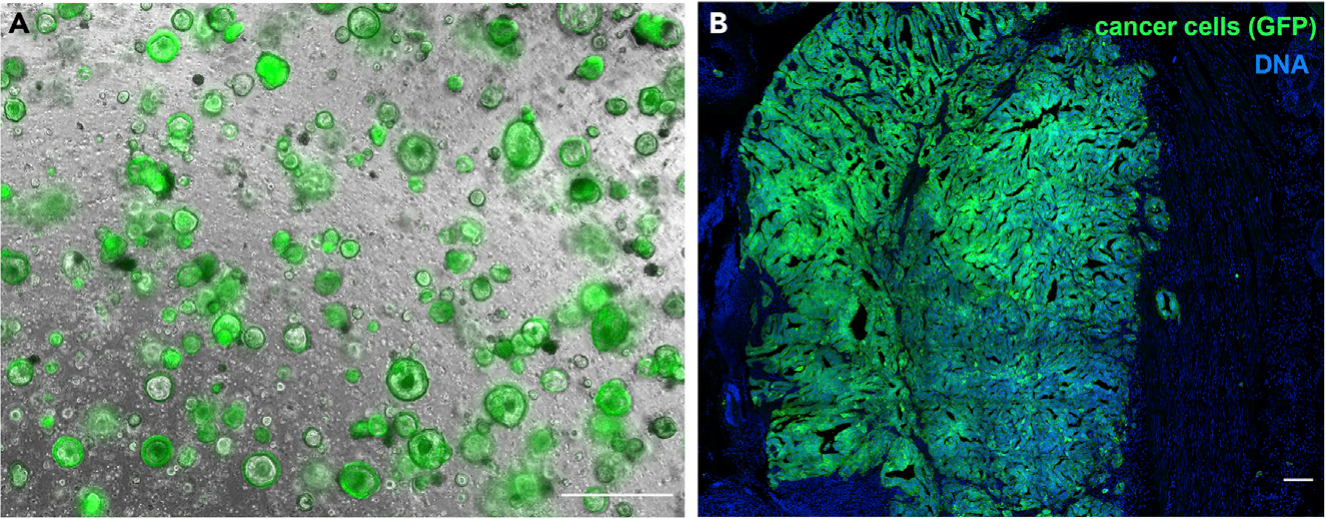
Collection of samples from mice injected with tumoroids (A) Tumoroids on the day of engraftment, highlighting the size and density of tumoroids. Scale bar, 500 μm. (B) Primary tumor developed in the colon of the mouse injected with mouse tumoroids. Cancer cells express GFP (green), nuclei of cells labeled with DAPI (blue). Scale bar 200 μm.

**Figure 6:**
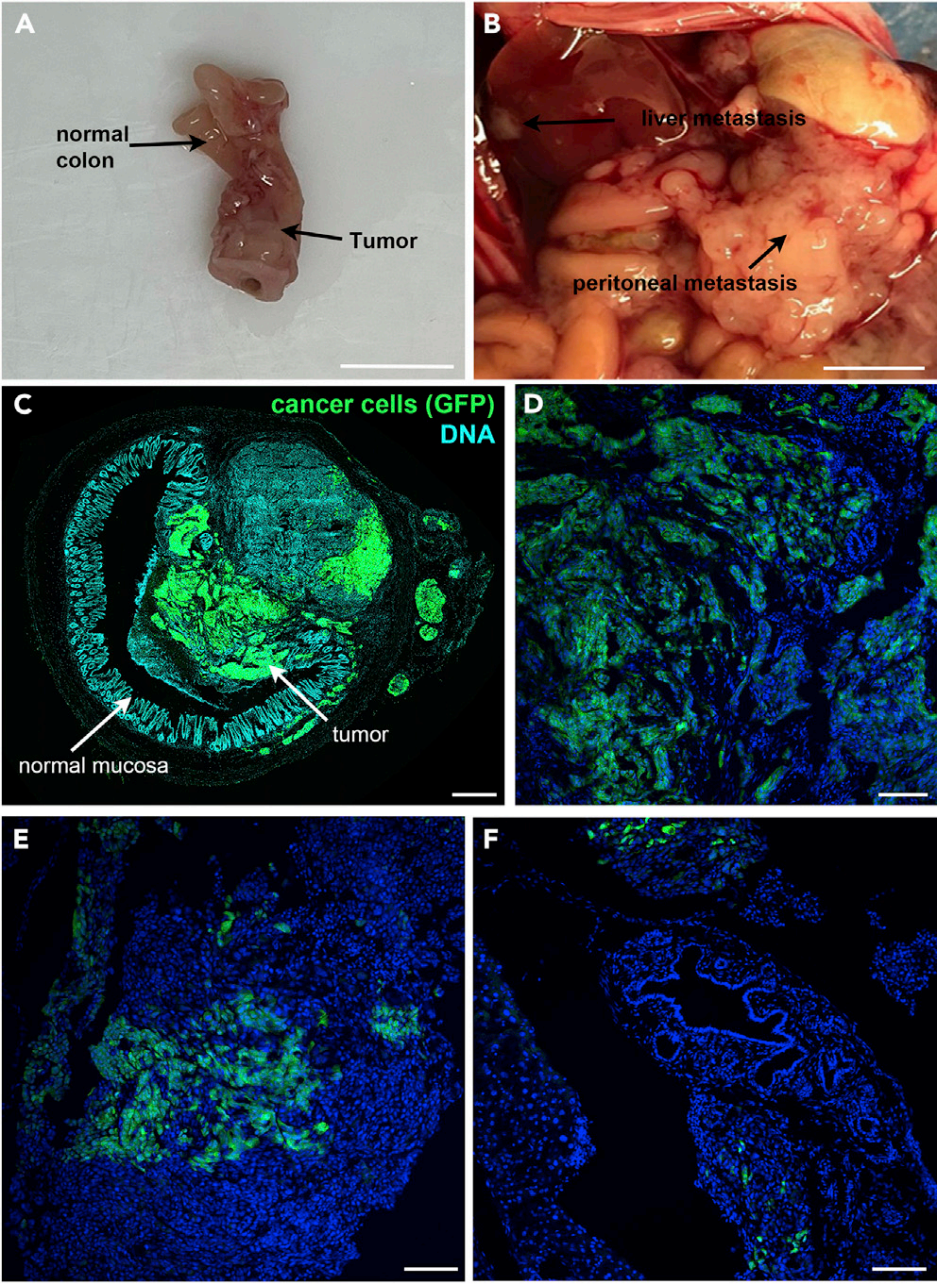
Collection of samples from mice injected with SW480GFP-LUC cancer cells (A) Colon of the mouse injected with SW480GFP-LUC cell line, exteriorized after 37 days. Scale bar, 10 mm. (B) The abdomen of the mouse injected with SW480GFP-LUC cells is inspected. Liver and peritoneal metastasis are observed. Scale bar, 1 cm. (C) Image of 10um-thick frozen colon section of a mouse injected with SW480GFP-LUC cancer cells. Cancer cells are detected by GFP signal (green), nuclei of cells labeled with DAPI (cyan). Scale bar 500 μm. (D) Higher magnification of a tumor developed in mice injected with SW480GFP-LUC cancer cells. Cancer cells express GFP (green), nuclei of cells labeled with DAPI (blue). Scale bar 100 μm. (E) Peritoneal metastasis developed in mice injected with SW480GFP-LUC cancer cells. Cancer cells express GFP (green), nuclei of cells labeled with DAPI (blue). Scale bar 100 μm. (F) Liver metastasis developed in mice injected with SW480GFP-LUC cancer cells. Cancer cells express GFP (green), nuclei of cells labeled with DAPI (blue). Scale bar 100 μm.

61. Image samples using an epifluorescent microscope or, for higher resolution images, a confocal microscope (Figures 5B and 6C–6F).

## Expected outcomes

### Analysis of the primary tumor and metastasis

Here we describe the results obtained with the tumoroids injected in Nude mice (Figure 5) colorectal cancer line SW480GFP-LUC and (Figure 6). In both cases, primary tumors developed in the distal colon, very close to the rectum (Figure 6A). During the autopsy, peritoneum and liver metastases were detected only in the mouse injected with SW480 cells (Figure 6B). To study the morphology of these tumors, 10 μm thick transversal sections of frozen tissue were made using a cryostat and deposited on slides. Nuclei were stained using DAPI, and the expression of GFP identified tumor cells. The entire transversal section of the colon was imaged using a LISPI confocal microscope (Figure 6C). Higher magnification images of the primary tumor and metastasis were taken using an LSM880 confocal microscope (Figures 6D–6F).

### Extension of the method to other cell lines and mouse strains

First, we tested if other mouse strains could be used with this procedure. We found that the Balb/c mouse strain is more sensitive to brush injury than C57BL/6 and Nude mice. While 50% of Balb/c mice (n = 16 mice) died one day after the procedure, the mortality rate was lower for other mice strains – only 21% of C57BL/6 (n = 102 mice) and 12% of Nude (n = 114 mice).

Second, we successfully used the protocol with other human colorectal cancer cell lines, such as HT29, HCT116, and SW837, injected in Nude mice, and mouse colon cancer cells, CT26 and MC38, injected in mice with matching genetic backgrounds: CT26 cells were injected in the Balb/c mouse strain, while the MC38 were injected in C57BL/6. Finally, we tested the protocol with primary human colon cancer cells isolated from patient-derived xenografts (PDX). We generated cell suspension by enzymatically dissociating PDX tumors, purifying live cells, and injecting them into Nude mice. While all models give rise to tumors, the efficiency of tumor development ranges from 25 to 100%.

Third, we tested if there is a relationship between the number of injected cells and the efficiency of tumor development. We injected either 0.5 or 2 million MC38 cells into C57BL/6 mice. We found that the higher number of injected cells leads to a higher percentage of mice that develop tumors. 2 million cells generated tumors in 89% of mice (n = 28), while 0.5 million cells generated tumoris in 31% of mice (n = 27).

Fourth, we assessed if the number of released crypts correlates with the efficiency of tumor development. We found that tumor development does not depend on the number of detached crypts for a high number of injected cells (2 × 10^6^ cells). However, for a lower number of injected cells (0.5 × 10^6^ cells), a higher percentage of tumors were obtained with 2,500 and 5,000 detached crypts (data for MC38 cells injected in C57BL/6 mice).

Fifth, we assessed the robustness of the protocol. As mentioned above, when injected with 2 million MC38 cancer cells, about 90% of mice developed tumors assessed by colonoscopy and confirmed by histological examination after necropsy. All mice needed to be sacrificed two weeks post-injection as tumors obstructed more than 50% of the colonic lumen. At that time, tumors were approximal the same size. If one wants to evaluate whether a specific gene product affects tumor initiation, that gene could be overexpressed or deleted in cancer cells before the injection. To calculate the number of animals required for each group, one can use the Fleiss formula.^6^^,^^7^ For example, if tumor incidence decreases to 50% and one would like to have an 80% chance of detecting this decrease, testing at p = 0.05, 14 animals per group are required. The tumor incidence when injecting 250 000 SW480 cells is 83%. Thus, using the same parameters, we calculate that 24 mice per group are required. Metastasis incidence in this model is 50% of mice. If incidence of metastasis increases to 80%, one would need 45 mice per group.

## Limitations

All tested cell types (human and mouse cell lines, dissociated tumoroids and cell suspension from PDX) can give rise to orthotopic tumors using this method. However, we noticed some limitations.

First, mouse genetic background may be a limiting factor. We found that the Balb/c mouse strain is sensitive to brush injury of colonic mucosa. Thus, we recommend the use of Nude and C57BL/6 mouse strains. Other strains must be tested for sensitivity to colonic injury before starting the protocol.

Second, metastasis development is inefficient. We observed metastasis only with SW480 cells injected in Nude mice. The lack of metastasis is likely due to the rapid growth of primary tumors. In most models, the primary tumors grow rapidly, reaching the size when the mouse must be sacrificed due to ethical reasons, which is insufficient time to establish metastasis.

Third, as it is hard to control the number of attached cells and as tumoroids coming from different mouse models could have different attachment abilities, it is hard to compare tumor growth using different tumoroids. Thus, tumor initiation, growth and metastasis could be only compared between the same parental cell lines or tumoroids. Imaging mice using IVIS one day after injection is important to ensure that the similar number of cells are grafted.

## Troubleshooting

### Problem 1

The colon is not cleaned of feces. Feces descend quickly and push the cells outward before they can attach to the wounded mucosa (steps 8 and 30).

### Potential solution

Massages of the mouse’s abdomen using fingers and pressures on the rectum must be performed to force the feces out of the colon. Wait for 10 min for all feces to exit.

If the colon is too full, which is felt under your fingers, proceed to the next mouse, while this one empties its colon naturally.

Do not wash the colon with PBS, as this dilutes the cell suspension.

***Note:*** We tried to deprive mice of food 12 h before injection. Even though this reduced the number of feces in the colon, the mice were too weak, and the percentage of mortality increased.

### Problem 2

The mouse rectum is still blocked after 4 h due to excessive glue (step 26).

### Potential solution

Gently remove the glue with a damp cloth, avoiding tearing the tissue.

Limit the amount of glue (around 20 μL–25 μL) when closing the anus.

### Problem 3

Sometimes, the growing tissue mass observed during endoscopy could be misinterpreted as a growing tumor. However, during microscopic analysis, this mass was identified as a local inflammation reaction due to the injury, not a tumor (step 33).

### Potential solution

Microscopic observation of the growing tissue mass is required to differentiate an inflammatory reaction from the tumor.

### Problem 4

High mouse mortality (step 16).

### Potential solution

High mortality for some mouse strains could be due to a bigger extent of the injury. As it is impossible to change the vibration speed on the electric interdental brush, the only possible solution could be to decrease the number and duration of manual injuries.

### Problem 5

The tumor grows too fast, obstructing the colon, and thus mouse needs to be sacrificed before metastases are developed (step 33).

### Potential solution

Decrease the number of cells to be injected.

## Resource availability

### Lead contact

Further information and requests for resources and reagents should be directed to and will be fulfilled by the lead contact: danijela.matic@curie.fr. All questions about the technical specifics of performing the protocol should be addressed to technical lead contact: sophie.richon@curie.fr.

### Materials availability

Only SW480GFPLUC cells were newly generated for this protocol, and they are available for the community. All other materials mentioned above are commercially available.

## Data Availability

This study did not generate datasets code.
